# Laparoscopic plug removal for chronic pain after inguinal hernia repair using the plug-and-patch technique: A case report

**DOI:** 10.1016/j.ijscr.2019.10.057

**Published:** 2019-10-28

**Authors:** Tatsuya Tazaki, Masaru Sasaki, Mohei Kohyama, Yoichi Sugiyama, Ryuta Shintakuya, Toshinori Hirano, Shinya Takahashi, Atsushi Nakamitsu

**Affiliations:** a Department of Surgery, JA Hiroshima General Hospital, Hiroshima, Japan; b Department of Surgery, Graduate School of Biomedical and Health Sciences, Hiroshima University, Hiroshima, Japan

**Keywords:** Chronic pain, Postoperative pain, Inguinal hernia, Case report

## Abstract

**Introduction:**

The use of tension-free mesh repair techniques for inguinal hernias has led to uniformly low recurrence rates. The main associated morbidity is chronic postoperative inguinal pain. Mesh removal and triple neurectomy is the indicated procedure; there is insufficient evidence to support mesh removal alone without neurectomy in patients with chronic postoperative inguinal pain.

**Presentation of case:**

A 76-year-old man previously underwent repair of a right inguinal direct hernia using the plug-and-patch technique. Two years later, he experienced groin pain requiring the use of pain medication. Five years after surgery, he expressed the desire to remove the mesh because of chronic pain, rated 8 out of 10 on a numeric rating scale. We suspected that he was experiencing nociceptive pain caused by a plug meshoma, so we performed a laparoscopic plug extraction. His inguinal pain improved to 2 out of 10 on the second postoperative day, and he stopped taking pain medication by 10 months after surgery.

**Discussion:**

The laparoscopic approach to plug removal is safe and simple. We successfully avoided causing new-onset pain by not using a groin incision to remove the mesh plug.

**Conclusion:**

Laparoscopic plug removal for nociceptive pain due to a plug meshoma is effective. However, since there is insufficient evidence to recommend mesh removal without triple neurectomy, informed consent and further consideration of techniques and diagnostic methods are needed.

## Highlights

• We must determine whether the plug or the onlay patch is the cause of chronic pain.
• Laparoscopic plug removal for nociceptive pain due to a plug meshoma is effective.
• We could avoid causing new-onset pain by not creating a groin incision.

## 1 Introduction

The main associated morbidity after tension-free mesh repair is chronic postoperative inguinal pain (CPIP) [1]. Surgery may be considered if CPIP does not improve with conservative treatment by a specialist for 6 months to 1 year—the time required for resolution of the inflammatory response triggered by the presence of mesh [[1], [2], [3]]. However, it is often difficult to determine when surgery is indicated. Triple neurectomy is the indicated procedure; there is insufficient evidence to support mesh removal alone without neurectomy in patients with CPIP [2]. Plug removal is necessary when the plug is causing nociceptive pain; the laparoscopic approach is easy to perform and provides adequate anatomic visualization without hindrance from previous scarring [4]. However, there are few reports [4] describing this procedure, and the questions remain whether to add an inguinal incision to remove the onlay patch and to perform triple neurectomy.

We report herein a patient who underwent laparoscopic plug removal for chronic pain after an inguinal hernia repair using the plug-and-patch technique. This study has been reported according to the Statement Updating Consensus Surgical Case Report (SCARE) guidelines [5].

## 2 Case presentation

A 76-year-old Japanese man previously underwent a right direct inguinal hernia repair using the plug-and-patch technique in our department. The PerFix™ Light Plug, size XL (C.R. Bard, Inc., Murray Hill, NJ), was fixed to the fascia transversalis using 4 absorbable sutures. The onlay patch was not sutured in place. The patient experienced severe groin pain after surgery, but this improved with administration of tramadol hydrochloride. Two years later, the patient again began to experience groin pain, for which he was prescribed pain medication at a nearby clinic.

**Fig. 1:**
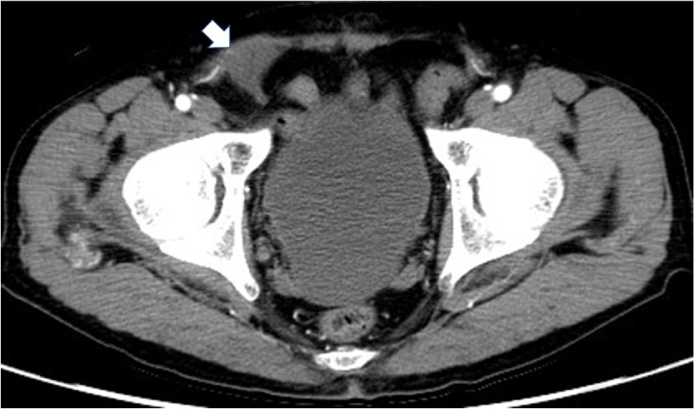
Abdominal computed tomography shows a meshoma at the location of the plug (white arrow).

Four years after the initial surgery, he visited our department with the complaint of right groin pain. A solid mass was palpable in the right inguinal region; we presumed this was attributable to the mesh. Palpation of the mass caused severe pain, but there was no pain at any other location. Abdominal computed tomography revealed a meshoma (Fig. 1). The patient was offered mesh extraction, but he declined. We consulted an anesthesiologist about the possibility of a nerve block and trigger point injections, but they advised that the effect would be temporary.

Five years after surgery, the patient expressed a desire for mesh removal, as his pain rated 8 out of 10 on a numeric rating scale. We determined that the likely etiology of his pain was nociceptive, caused by a plug meshoma. Computed tomography findings indicated that the plug would be easy to extract by a transabdominal surgical approach. We thought there was a high possibility of improving the patient’s pain by removing the plug, and laparoscopic surgery would be an easy way to accomplish this. However, the International Guidelines for Groin Hernia Management (IGGHM) recommend mesh removal with triple neurectomy for patients with CPIP [2]. In addition to laparoscopic plug removal, we also considered removing the onlay patch and performing triple neurectomy at the same time, but in our patient with mainly nociceptive pain caused by the plug, we felt the more extensive procedure would be overly invasive and would carry the risk of causing new pain by adding a groin incision. We offered the patient laparoscopic plug extraction with simultaneous resection of the genital branch of the genitofemoral nerve. If this procedure did not improve his pain, the plan was to later remove the onlay patch and perform iliohypogastric and ilioinguinal nerve resection by an anterior approach. The above explanation was given to the patient and consent was obtained.

**Fig. 2:**
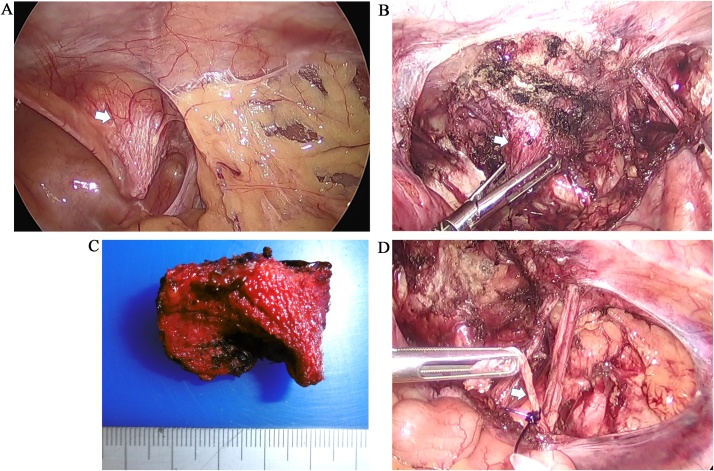
Operative findings. (a) Laparoscopy revealed that the plug projected into the abdominal cavity (white arrow). (b) The plug (white arrow), projecting on the medial side of the inferior epigastric artery, is excised. (c) The extracted plug. (d) The genital branch of the genitofemoral nerve (white arrow) is ligated and dissected.

Laparoscopy revealed that the plug projected into the abdominal cavity (Fig. 2a). The plug was excised using an ultrasonically activated device (Fig. 2b, c). In addition, the genital branch of the genitofemoral nerve was exposed, ligated using an ENDLOOP® Ligature (Ethicon, USA), and dissected (Fig. 2d). Finally, the peritoneum was closed using continuous suturing technique.

The patient reported that his inguinal pain improved to 2 out of 10 on the second postoperative day, and he was discharged from the hospital. He still required pain medication for low back pain, but he stopped taking this by 10 months after surgery. There has been no recurrence of the inguinal hernia.

## 3 Discussion

The International Guidelines for Groin Hernia Management (IGGHM) define CPIP as moderate pain that affects daily activities and lasts for longer than 3 months postoperatively [2]. The reported incidence of CPIP ranges from 0.7% to 43.3%; the reason for this large difference is because of different times of assessment and different methods of measurement in the existing reports [6]. Between 0.5% and 6% of patients experience debilitating pain affecting normal daily activities or work [7]. While the IGGHM considers CPIP to be primarily of neuropathic origin [2], it remains controversial whether CPIP is more likely to be nociceptive or neuropathic. Nociceptive pain is caused by chronic, continuous stimulation of pain fibers by compression resulting from excessive fibrosis associated with wrinkling of the mesh (meshoma).

Globally, the most commonly performed surgery for tension-free repair of inguinal hernias is the Lichtenstein method [8], which is one of the techniques recommended by the IGGHM [2]. However, the plug-and-patch technique [9] is preferred in Japan, where CPIP seems to most frequently occur in patients who underwent this technique.

An international consensus algorithm recommends triple neurectomy by a herniologist when neuropathic pain is not improved by conservative treatment by a pain team. If a meshoma is determined to be the cause of the pain, resection is recommended. Since neuropathic pain often coexists with nociceptive pain caused by a meshoma, and there is a possibility that new nerve damage may occur while removing the mesh, simultaneous nerve resection at the time of mesh removal is recommended [2]. The IGGHM states that there is insufficient evidence to support mesh removal alone without neurectomy in patients with CPIP, and that there is insufficient evidence for the diagnostic and therapeutic value of nerve blocks [2]. The trigger point injection is a simple procedure that can be performed by a general surgeon. We did not perform it with the advice of an anesthesiologist. However, if it could be effective temporarily, it might have been done for our patient who did not want surgery at first.

Our patient’s plug was considered amenable to the laparoscopic approach. Laparoscopic surgery can also confirm the absence of hernia recurrence, and there are rare case reports of pain improvement with laparoscopic plug removal alone [4]. The procedure was easy because we were sufficiently experienced with the transabdominal preperitoneal (TAPP) method and had experience in laparoscopic plug removal for hernia recurrence [10]. We considered removing the onlay patch with triple neurectomy, but we decided against these procedures because of the risk of causing additional pain by creating an inguinal incision. If the patient had not experienced adequate improvement in symptoms, we planned to adopt the latter approach as a second phase of pain-relief surgery. It is important to note that a 2-stage plan should not be selected if the patient is unwilling to undergo a second surgery. In addition to laparoscopic plug removal, we also resected the genital branch of the genitofemoral nerve; this can easily be performed in the same field of view. However, this procedure may have been unnecessary, as our patient reported no scrotal pain.

When a patient has previously undergone a plug-and-patch hernia repair, it is necessary to determine whether the plug or the onlay patch is the cause of CPIP. It is necessary to examine the effectiveness of trigger point injection for diagnosis and treatment and to accumulate evidence. When it is determined that a patient has nociceptive pain due to a plug, laparoscopic plug removal alone may be adequate. If the operator is familiar with the TAPP method, the procedure is simple and provides good anatomic visualization without hindrance from previous scarring. We were able to successfully avoid causing new-onset pain for our patient by not creating a groin incision at the time of plug removal.

## 4 Conclusion

In patients who experience plug-related nociceptive CPIP after a hernia repair using the plug-and-patch technique, laparoscopic plug removal is a simple and safe method. Further research into techniques, including diagnostic methods, is necessary.

## Sources of funding

This research did not receive any specific grant from any funding agency.

## Ethical approval

The approval of our institutional ethics committee is unnecessary for a clinical case report.

## Consent

Informed consent was obtained from the patient for publication of this case report and accompanying images.

## Author’s contribution

Study conception and design: Tazaki.

Surgical team: Kohyama, Sugiyama, Shintakuya, Hirano.

Critical revision of manuscript: Sasaki, Takahashi, Nakamitsu.

All authors have read and approved the final manuscript.

## Registration of research studies

This case report was not registered.

## Guarantor

Tatsuya Tazaki.

## Provenance and peer review

Not commissioned, externally peer-reviewed.

## Declaration of Competing Interest

All authors have no conflicts of interest.
